# Determinants of spring migration departure decision in a bat

**DOI:** 10.1098/rsbl.2017.0395

**Published:** 2017-09-20

**Authors:** Dina K. N. Dechmann, M. Wikelski, D. Ellis-Soto, K. Safi, M. Teague O'Mara

**Affiliations:** 1 Department of Immuno-ecology and Migration, Max Planck Institute for Ornithology, 78315 Radolfzell, Germany; 2 Department of Biology, University of Konstanz, 78464 Konstanz, Germany

**Keywords:** Chiroptera, migration onset, *Nyctalus noctula*

## Abstract

Migratory decisions in birds are closely tied to environmental cues and fat stores, but it remains unknown if the same variables trigger bat migration. To learn more about the rare phenomenon of bat migration, we studied departure decisions of female common noctules (*Nyctalus noctula*) in southern Germany. We did not find the fattening period that modulates departure decisions in birds. Female noctules departed after a regular evening foraging session, uniformly heading northeast. As the day of year increased, migratory decisions were based on the interactions among wind speed, wind direction and air pressure. As the migration season progressed, bats were likely to migrate on nights with higher air pressure and faster tail winds in the direction of travel, and also show high probability of migration on low-pressure nights with slow head winds. Common noctules thus monitor complex environmental conditions to find the optimal migration night.

## 1. Background

Migration is a fascinating animal behaviour, and yet we still lack the most basic information, especially from non-model organisms [1–3]. While over 30% of Palaearctic and Antarctic birds migrate long distances [4], only a few of the more than 1300 bat species have maximum migration distances of over 1000 km [5,6]. Understanding this migration is important not only from an evolutionary viewpoint but also in light of vast numbers of casualties caused by wind turbines, particularly among long-distance migrants [7–9].

There are profound differences between the life cycles of birds and migrating bats. In European temperate bats, females primarily execute long-distance northeasterly migrations to insect-rich regions [6,8]. After hibernation, they begin gestation and are under considerable time pressure to raise offspring having travelled hundreds of kilometres to the same maternity colony each year. Females then return southwest in the autumn to their overwintering habitats where they mate, and prepare for and enter hibernation. The correct timing of migration is critical for these females, as they must balance accumulating fat reserves to help fuel migration against load carrying as gestation proceeds. Bats should also time their migration departure to take advantage of environmental conditions [10] conducive to long flights (i.e. over 100 km, [11]) and arrive at maternity colonies when insect abundance begins to rise.

Migration onset in birds is primarily triggered by date and environmental variables including (i) physiological preparation, especially fattening, (ii) day length, (iii) suitable wind conditions (low or tailwind), and (iv) atmospheric pressure [3,12]. However, most of our knowledge comes from stopover sites [13]. Female common noctules rapidly and easily gain weight after hibernation [11] and migrating pipistrelle bats use a mix of ingested food and stored fat to fuel their short migration steps [14], reflecting a fundamental difference in the physiological constraints of bat and bird migration. In noctules, migration quickly follows hibernation, and should rely heavily on environmental changes that indicate both seasonal changes and advantageous flying conditions. Previous work indicates that similar to birds, bats use environmental variables to make migration decisions, choosing low wind speeds when crossing the North Sea [15]. However, it is unknown which environmental cues bats use when making the decision to depart. A predictive model like the one we present here, based on data from a multi-year telemetry study on the common noctule (*Nyctalus noctula*), can provide useful insights to target tracking periods and for wind power planning [7,8].

## 2. Material and methods

We captured female common noctules (*N. noctula*) in 2012, 2013 and 2016 at two sites. In the Seeburgpark Kreuzlingen, Switzerland (47.649928° N, 9.186123° E), bats were captured between 11 and 14 h from bat boxes, and from the roof of the Reichenau-Waldsiedlung school (47.696738° N, 9.117721° E) at emergence (20–21 h) [11]. We weighed bats (±0.5 g) and measured their forearm length (±0.1 mm). We marked bats with a subcutaneous pit-tag (ID100; Euro ID, Weilerswist, Germany), equipped them with external radio-transmitters (for details see [11]) and tracked them using wide range telemetry receivers (AR8000/8200, AOR Ltd; Sika, Biotrack) with collapsible H- or Yagi-antennas. We released all bats within an hour at the site of capture.

In the mornings one of us (MW) searched in an airplane in all directions (Cessna 172; [11]) to determine which bats had left for migration and their migration direction. Maximum detection range of the radio signals was approximately 7.5 km. Migration data are available at the Movebank Data Repository ([11], doi:10.5441/001/1.f01815nq).

We obtained hourly measurements for all weather variables from Konstanz (weather station 2712; see also electronic supplementary material 1, figure S1) through the German Meteorological Office during our study as well as long-term weather. We used measurements collected nightly at 21 h as estimates of environmental conditions, around the time when bats were likely making migration decisions. There was an increase in day length of 1.5 h over the course of the migratory departure period at our site.

We fitted logistic generalized linear mixed effects models with bat identity as a random effect in *lme4* [16]. Each day that the bat was present in the research site, including the day of capture, was scored as 0 (no migration) or 1 (day of migration). We then used two sets of models to evaluate migration timing. The first used a likelihood ratio test to evaluate if body condition at capture (body mass/forearm length, a standard measure of size for bats) influences the time to migration and therefore if the bats need a set amount of time to increase post-hibernation body condition prior to migration. In the second step, we used a model selection approach to test environmental effects (numerical day of the year, air temperature, wind direction, wind speed, air pressure and relative humidity) on the likelihood of departure. Following Zurr *et al*. [17], we found no temporal (daily) autocorrelation in the data for any of the weather variables. Because of large differences in absolute values among environmental variables we rescaled all variables to have a centre of 0. We created stepwise reductions in model complexity from a full interaction and additive-only model, by removing fixed effects with the lowest *Z*-values in each model (table 1; electronic supplementary material 2). We calculated the Akaike information criterion corrected for small sample sizes (AICc) and change in AICc (ΔAICc), as well as the conditional and marginal *R*^2^ for each model in *MuMIn* [18–20]. All analyses were performed in R 3.3.2.

**Table 1.** Top performing models describing noctule migration departure all contain variable interactions instead of the variables themselves. ΔAICc notes the change in Akaike information criterion corrected for small sample sizes from an interaction model. See electronic supplementary material 2 for full model summaries. Bat ID was always added as random effect.

model	AICc	ΔAICc	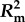	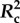
day of year + wind direction × wind speed + wind speed × air pressure + wind direction × wind speed × air pressure	163.44	−24.49	0.3	0.66
day of year + wind direction + day of year × wind direction + wind direction × wind speed + wind speed × air pressure + day of year × wind speed × air pressure	164.14	−23.79	0.31	0.68
day of year + wind direction + wind speed + day of year × wind direction + wind direction × wind speed + wind speed × air pressure + day of year × wind speed × air pressure	164.79	−23.14	0.25	0.58
day of year + day of year × wind direction + wind direction × wind speed + wind speed × air pressure + day of year × wind speed × air pressure	165.06	−22.87	0.24	0.57
day of year + wind direction + wind speed + day of year × wind direction + wind direction × wind speed + wind speed × air pressure + day of year × wind direction × wind speed + day of year × wind speed × air pressure	165.32	−22.61	0.37	0.73

## 3. Results

**Figure 1.**
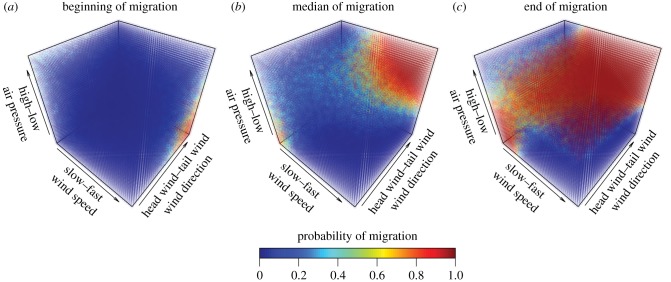
Predicted interaction probabilities for noctule migratory departure from the best AICc model at the beginning, median and end dates of the spring migration season.

**Figure 2.**
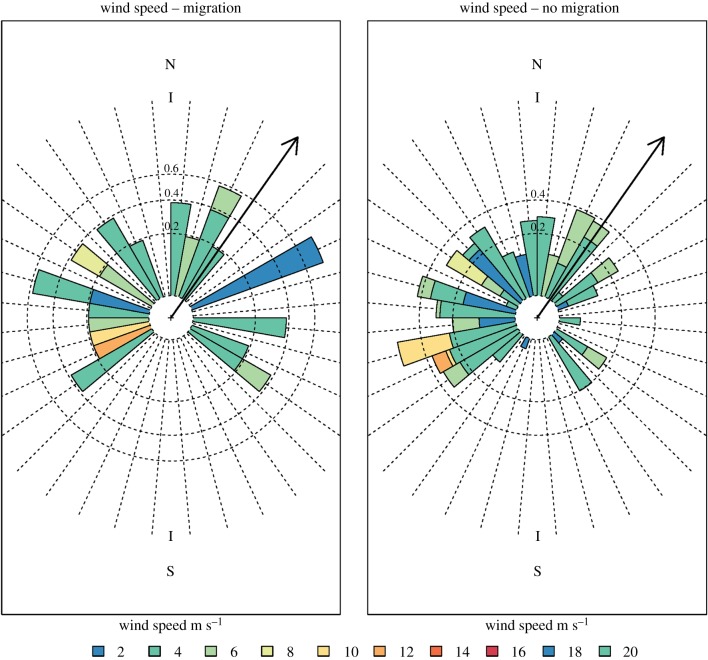
(*a*) Wind speed and direction from which the wind was blowing during nights when bats migrated, and (*b*) nights when they did not. Black arrow: mean migration direction. See also [11].

We tracked 29 females for 1–22 nights (6.4 ± 5.5; mean ± s.d.) before they left our study area. We found no effect of body condition on the number of days until departure (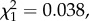 *p* = 0.845; electronic supplementary material 1, figure S2). The best performing environmental models included increasing numerical day of year and several iterations of the interactions of wind direction, wind speed, and air pressure (table 1; electronic supplementary material 2). As these factors interacted across the migration period, bats were more likely to migrate on nights with faster tail winds, beginning at the median wind speed of 2 m s^−1^, particularly on nights with higher air pressure (figure 1). However, these conditions were not absolute and several bats chose to migrate in slow headwind conditions on nights with low air pressure (figure 1*b*, lower left corner). Additionally, a circular Watson–Williams test for homogeneity of means found no difference in mean wind direction (*W*_2_ = 2.060, *p* = 0.357, figure 2) or wind speed (*W*_2_ = 3511, *p* = 0.462) on migratory versus non-migratory nights when treated as single factors, reinforcing that bats assess interactions when making their decisions (electronic supplementary material 1, figure S1 for distribution of weather data in study period; electronic supplementary material 1, figure S3 for year-round wind data).

## 4. Discussion

In contrast with songbirds [13,17,21], the need to increase body condition prior to migration had little influence on departure timing in common noctule bats. Importantly, it was the interaction among wind speed, direction and air pressure that when scaled by day of the year yielded the best fit for estimating the probability that noctules would migrate. The comparison between the ‘songbird migration model’ and our bats thus has important implications for our understanding of migration in small aerial vertebrates as it adds an unexpected dimension to understanding how migratory animals gauge environmental conditions. The use of combinations of weather variables provides a stable environmental cue whereby bats can choose optimal conditions to migrate early in the season (fast tail winds on low-pressure nights) and when faced with deteriorating weather conditions they tend to prioritize fast tail winds.

The difference in the distance of the migration steps executed by birds and bats may allow for the nearer-term migration strategies used by bats. Many birds spend extended time periods fattening before executing migration steps that span hundreds to thousands of kilometres [13]. Non-hibernating bats store fat for short-term use [14], which may fuel a limited aspect of their relatively shorter migration flights [10,22]. Common noctules appear to stop over frequently to forage and refuel [11], and unlike most birds, they may use daytime torpor during stop overs to minimize daily energy use [10,23]. Noctules foraged during the first 90 min after sunset at this study site, ingesting up to 30% of their mass [11,24]. This pattern was independent of whether bats migrated later that night [11]. They then likely powered flight from both recently ingested food and body stores [14,25], a strategy that maximizes food intake and the time available for migration [22,26].

Noctules are clearly capable of monitoring subtle but complex changes in weather conditions and use the interaction among wind speed and direction and air pressure to decide when to migrate. The few bats at the beginning of the migration season left only on low-pressure nights with fast tail winds. This gradually shifted to an increased probability to leave on either lowpressure nights with slow head winds (calm, clear weather) or nights with high air pressure and fast tail winds. High air pressure indicates good weather conditions, which the bats appear to prefer. This emphasizes the flexibility that bats show in their migration strategies and that there is no single best set conditions for female noctules when deciding to leave. Future work should target the social or physiological sources of the variation in this flexibility to resolve the many iterations in migratory strategies found in birds and bats.

Migration appears to be a balance of trade-offs that maximizes immediate foraging returns along short migration steps toward northeastern maternity colonies. Our results show that migration onset in bats reflects a multivariate strategy that differs from the better-known songbird model. Migration, which has evolved several times in bats, needs further study to understand the seasonal and environmental impacts on animal movement across the planet [27].

## Supplementary Material

Supplementary figures

## Supplementary Material

Model selection

## Supplementary Material

Data file

## Supplementary Material

Model selection, including discarded models

## Data accessibility

Migration data are available at the Movebank Data Repository: http://dx.doi.org/10.5441/001/1.f01815nq [11]. All other data are available in the electronic supplementary material.
