# Supplementary material for: Determinants of spring migration departure decision in a bat
Source: Biol Lett. 2017 Sep 20;13(9):20170395. doi: 10.1098/rsbl.2017.0395 (PMC5627173; doi:10.1098/rsbl.2017.0395)
Supplement: Supplementary figures [file rsbl20170395supp1.docx]

Electronic Supplementary Material 1. Supplementary figures.

Determinants of spring migration departure decision in a bat

Dina K.N. Dechmann, M. Wikelski, D. Ellis-Soto, K. Safi, M. Teague O'Mara

Figure S1. Density distributions of the weather data collected on nights when bats migrated (red shading) and did not migrate (grey shading) used in the model selection process.

Figure S2: Body condition (mass / forearm length) of female *N. noctula* at capture and the number of days spent at the study site after capture before migration onset. There is no indication that bats are limited in their decision by the need to increase body condition prior to migration.

Figure S3. Historical data for wind speed and direction in Konstanz, Germany for the common noctule spring migration season in April and May. The arrow indicates the general direction of migration travel during our tracking periods.
