# Supplementary material for: Determinants of spring migration departure decision in a bat
Source: Biol Lett. 2017 Sep 20;13(9):20170395. doi: 10.1098/rsbl.2017.0395 (PMC5627173; doi:10.1098/rsbl.2017.0395)
Supplement: Model selection [file rsbl20170395supp2.docx]

Electronic Supplementary Material 2 – Model selection.

Determinants of spring migration departure decision in a bat

Dina K.N. Dechmann, M. Wikelski, D. Ellis-Soto, K. Safi, M. Teague O'Mara

**Model selection process.** Based on our 285 observations of 29 bats and the large number of possible interactions among the weather variables (day of year, wind direction, wind speed, air pressure, air temperature, relative humidity), we first searched for an initial model based on the combinations of only five initial parameters. We chose the initial model that had the lowest AICc and then conducted a stepwise reduction in model complexity by removing the parameter that has the lowest absolute Z-score value and then refitting the model. From this we had 6 best fit reduced models that were within 2 AICc units of the best-ranked model.

Supplemental Table 1. Initial binomial generalized linear mixed effects models and the reduced models evaluated through model selection and their difference in AICc (∆AICc) from the first interaction model. Marginal (R^2^_m_ – fixed effects only), and conditional (R^2^_c_ – fixed + random effects) pseudo R^2^ and AICc are calculated for each model using *MuMIn*. The best performing reduced models 2 AICc are noted in italics. Bat ID was always added as random effect.

| **Initial models** | **AICc** |  | **R^2^_m_** | **R^2^_c_** |
| --- | --- | --- | --- | --- |
| day of year*wind direction*wind speed*air pressure*air temperature | 187.93 |  | 0.99 | 0.99 |
| day of year*wind direction*wind speed*air pressure*relative humidity | 194.16 |  | 0.95 | 0.98 |
| day of year*wind direction*wind speed*air temperature* relative humidity | 195.08 |  | 0.90 | 0.95 |
| day of year*wind direction*air pressure*air temperature* relative humidity | 198.80 |  | 0.87 | 0.88 |
| day of year*wind speed*air pressure*air temperature* relative humidity | 198.92 |  | 0.78 | 0.87 |
| wind direction*wind speed*air pressure*air temperature* relative humidity | 203.83 |  | 0.69 | 0.78 |
| **Best fit reduced models** | **AICc** | **∆AICc** | **R^2^_m_** | **R^2^_c_** |
| *day of year + wind direction*wind speed + wind speed*air pressure + wind direction*wind speed*air pressure* | *163.44* | *-24.49* | *0.3* | *0.66* |
| *day of year + wind direction + day of year*wind direction + wind direction*wind speed + wind speed*air pressure + day of year*wind speed*air pressure* | *164.14* | *-23.79* | *0.31* | *0.68* |
| *day of year + wind direction + wind speed + day of year*wind direction + wind direction*wind speed + wind speed*air pressure + day of year*wind speed*air pressure* | *164.79* | *-23.14* | *0.25* | *0.58* |
| *day of year + day of year*wind direction + wind direction*wind speed + wind speed*air pressure + day of year*wind speed*air pressure* | *165.06* | *-22.87* | *0.24* | *0.57* |
| *day of year + wind direction + wind speed + day of year*wind direction + wind direction*wind speed + wind speed*air pressure + day of year*wind direction*wind speed + day of year*wind speed*air pressure* | *165.32* | *-22.61* | *0.37* | *0.73* |
| **Additional reduced models** |  |  |  |  |
| day of year + wind direction*wind speed + wind speed*air pressure + wind direction*wind speed*air pressure | 165.69 | -22.24 | 0.24 | 0.66 |
| day of year + wind direction + wind speed + day of year*wind direction + wind direction*wind speed + day of year*air pressure + wind speed*air pressure + day of year*wind direction*wind speed + day of year*wind speed*air pressure | 166.08 | -21.85 | 0.39 | 0.74 |
| day of year + wind direction + wind speed + day of year*wind direction + day of year*wind speed + wind direction*wind speed + day of year*air pressure + wind speed*air pressure + day of year*wind direction*wind speed + day of year*wind direction*air pressure + day of year*wind speed*air pressure | 167.36 | -20.57 | 0.43 | 0.76 |
| day of year + wind direction*wind speed + wind speed*air pressure | 169.28 | -18.65 | 0.23 | 0.59 |
| wind direction*wind speed + wind speed*air pressure + wind direction*wind speed*air pressure | 169.69 | -18.24 | 0.18 | 0.52 |
| day of year + wind direction*wind speed + day of year*wind speed*air pressure | 169.81 | -18.12 | 0.23 | 0.56 |
| day of year + day of year*wind speed*air pressure | 171.27 | -16.66 | 0.19 | 0.53 |
| day of year + air pressure | 173.43 | -14.5 | 0.22 | 0.61 |
| day of year + relative humidity + air pressure | 173.87 | -14.06 | 0.22 | 0.61 |
| day of year + relative humidity + air pressure + wind speed | 174.33 | -13.6 | 0.23 | 0.64 |
| wind speed*air pressure + wind speed*air pressure*wind direction | 174.64 | -13.29 | 0.14 | 0.51 |
| day of year + air pressure + wind speed | 174.9 | -13.03 | 0.22 | 0.63 |
| day of year | 176.5 | -11.43 | 0.15 | 0.54 |
| day of year + wind direction + air pressure + wind speed | 176.62 | -11.31 | 0.22 | 0.61 |
| day of year + wind speed*air pressure | 177.4 | -10.53 | 0.16 | 0.55 |
| air temperature + day of year + wind direction + air pressure + wind speed | 178.69 | -9.24 | 0.22 | 0.62 |
| wind speed*air pressure*wind direction | 183.66 | -4.27 | 0.03 | 0.36 |
| air pressure | 185.39 | -2.54 | 0.02 | 0.33 |
| day of year + wind direction*wind speed + wind speed*air pressure + wind direction*wind speed*air pressure | 165.69 | -22.24 | 0.24 | 0.66 |
| day of year + wind direction + wind speed + day of year*wind direction + wind direction*wind speed + day of year*air pressure + wind speed*air pressure + day of year*wind direction*wind speed + day of year*wind speed*air pressure | 166.08 | -21.85 | 0.39 | 0.74 |
| day of year + wind direction + wind speed + day of year*wind direction + day of year*wind speed + wind direction*wind speed + day of year*air pressure + wind speed*air pressure + day of year*wind direction*wind speed + day of year*wind direction*air pressure + day of year*wind speed*air pressure | 167.36 | -20.57 | 0.43 | 0.76 |
| day of year + wind direction*wind speed + wind speed*air pressure | 169.28 | -18.65 | 0.23 | 0.59 |
| wind direction*wind speed + wind speed*air pressure + wind direction*wind speed*air pressure | 169.69 | -18.24 | 0.18 | 0.52 |
| day of year + wind direction*wind speed + day of year*wind speed*air pressure | 169.81 | -18.12 | 0.23 | 0.56 |
| day of year + day of year*wind speed*air pressure | 171.27 | -16.66 | 0.19 | 0.53 |
| day of year + air pressure | 173.43 | -14.5 | 0.22 | 0.61 |
| day of year + relative humidity + air pressure | 173.87 | -14.06 | 0.22 | 0.61 |
| day of year + relative humidity + air pressure + wind speed | 174.33 | -13.6 | 0.23 | 0.64 |
| wind speed*air pressure + wind speed*air pressure*wind direction | 174.64 | -13.29 | 0.14 | 0.51 |
| day of year + air pressure + wind speed | 174.9 | -13.03 | 0.22 | 0.63 |
| day of year | 176.5 | -11.43 | 0.15 | 0.54 |
| day of year + wind direction + air pressure + wind speed | 176.62 | -11.31 | 0.22 | 0.61 |
| day of year + wind speed*air pressure | 177.4 | -10.53 | 0.16 | 0.55 |
| air temperature + day of year + wind direction + air pressure + wind speed | 178.69 | -9.24 | 0.22 | 0.62 |
| wind speed*air pressure*wind direction | 183.66 | -4.27 | 0.03 | 0.36 |
