# Supplementary figures and images for: Determinants of spring migration departure decision in a bat
Source: Biol Lett. 2017 Sep 20;13(9):20170395. doi: 10.1098/rsbl.2017.0395 (PMC5627173; doi:10.1098/rsbl.2017.0395)

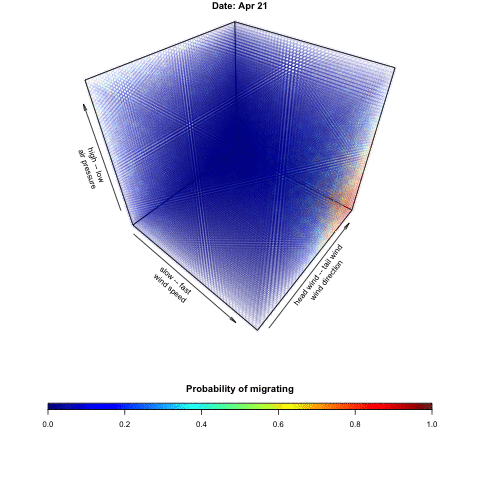

Supplement: Model selection, including discarded models [file rsbl20170395supp4.gif]
